# The correlation between baseline score and post-intervention score, and its implications for statistical analysis

**DOI:** 10.1186/s13063-018-3108-3

**Published:** 2019-01-11

**Authors:** Lei Clifton, David A. Clifton

**Affiliations:** 1 Centre for Statistics in Medicine (CSM), NDORMS, University of Oxford, Oxford, UK; 2 Institute of Biomedical Engineering (IBME), Department of Engineering Science, University of Oxford, Oxford, UK

**Keywords:** Correlation, Baseline, Change score, Post-intervention, Treatment, Statistical analysis, Sample size, Independent, Means, Standard error (SE), Standard deviation (SD), Regression to the mean (RTM), Outcome, Analysis of covariance (ANCOVA), Randomised controlled trial (RCT), Bland-Altman plot

## Abstract

**Background:**

When using a continuous outcome measure in a randomised controlled trial (RCT), the baseline score should be measured in addition to the post-intervention score, and it should be analysed using the appropriate statistical analysis.

**Methods:**

We derive the correlation between the change score and baseline score and show that there is always a correlation (usually negative) between the change score and baseline score. We discuss the following correlations and provide the mathematical derivations in the Appendix:

- Correlation between change score and baseline score
- Correlation between change score and post score
- Correlation between change score and average score.

The setting here is a parallel, two-arm RCT, but the method discussed in this paper is applicable for any studies or trials that have a continuous outcome measure; it is not restricted to RCTs.

**Results:**

We show that using the change score as the outcome measure does not address the problem of regression to the mean, nor does it take account of the baseline imbalance. Whether the outcome is change score or post score, one should always adjust for baseline using analysis of covariance (ANCOVA); otherwise, the estimated treat effect may be biased. We show that these correlations also apply when comparing two measurement methods using Bland-Altman plots.

**Conclusions:**

The correlation between baseline and post-intervention scores can be derived using the variance sum law. We can then use the derived correlation to calculate the required sample size in the design stage. Baseline imbalance may occur in RCTs, and ANCOVA should be used to adjust for baseline in the analysis stage.

## Background

When using a continuous outcome measure in a randomised controlled trial (RCT), the baseline score should be measured in addition to the post-intervention score. In a previous paper [9], we have shown how to derive *r*, the correlation between baseline score and post score. In this paper, we derive correlations between different variables in the Appendix, assuming *r* is known. There are two options for the outcome measure: change score or post score. We examine the validity of using change score as the outcome measure in the “Method” section, and then further discuss the applications of our methods outlined in the “Discussion” section.

## Method

### The choice of outcome measure: post score vs. change score

The methods outlined in this paper are suitable for any continuous measure, and therefore we have used a generic notation. Suppose the primary continuous outcome measure is *Y*, with *Y*_0_ and *Y*_1_ denoting the value of *Y* at baseline and post-intervention, respectively. Let *r* denote the correlation coefficient between *Y*_0_ and *Y*_1_.

We will call *Y*_1_ “post score”, *Y*_0_ “baseline score”, and (*Y*_1_ − *Y*_0_) “change score”. We note that *Y*_1_ is also called “follow up score” in [17]. The authors assumed there is no interaction between baseline and intervention group, and we make the same assumption in this paper.

In analysis of covariance (ANCOVA), we estimate parameters *a*, *b*, and *c* in the following regression question:

$$ \mathrm{post}\ \mathrm{score}=a+b\times \mathrm{baseline}+c\times \mathrm{group} $$

where “group” stands for “intervention group”. One is usually most interested in the estimate of *c*, the treatment effect, in an RCT. Substituting *Y*_1_ for “post score”, *Y*_0_ for “baseline score”, and *G* for “group”, we have the following regression equation for a standard ANCOVA that uses post score as outcome:

1 $$ {Y}_1=a+b{Y}_0+ cG $$

Rearranging Eq. 1, we have

2 $$ {Y}_1-{Y}_0=a+\left(b-1\right){Y}_0+ cG $$

Equation 2 is ANCOVA using change score (*Y*_1_ − *Y*_0_) as outcome and adjusting for baseline *Y*_0_. Compared with the standard ANCOVA in Eq. 1, where post score *Y*_1_ is the outcome, nothing has changed except that the regression coefficient for *Y*_0_ has decreased by 1. The significance level and the width of the confidence intervals for all estimated regression coefficients remain the same as those in a standard ANCOVA. Further mathematical details can be found in [14].

Rearranging Eq. 1 in a different way, we have

3 $$ {Y}_1-b{Y}_0=a+ cG $$

Equation 3 shows that using change score as outcome without adjusting for baseline is only equivalent to a standard ANCOVA when *b* = 1. In practice, the estimated *b* in an ANCOVA is rarely equal to 1; hence, it is only a special case of ANCOVA.

### Regression to the mean (RTM) and ANCOVA

RTM is a well-known statistical phenomenon, first discovered by Galton in [10]. RTM has been discussed by a number of authors, e.g. [3, 6, 7, 15], etc. In this paper, we consider RTM in the context of baseline measures.

If an extreme measure is observed at baseline, then its value is likely to be less extreme in the post-intervention measure, even if the intervention has no effect. In the RCT example in [17], the treatment effect of acupuncture was measured by a 100-point rating score, where lower scores indicate poorer outcomes. Suppose that the baseline scores of the control group reflect the scores of the general population, and that acupuncture has no treatment effect. If, by chance, the baseline scores of the intervention group are lower than the scores of the general population, their post scores will still be higher than their baseline scores, due to RTM. We consider two options of outcome measure:

1. Post score: If post score is positively correlated with the baseline score (which is usually the case in clinical practice), acupuncture will appear to have a negative effect, even though it has no effect; i.e. the treatment effect of acupuncture will be under-estimated.
2. Change score: Acupuncture will appear to have a positive effect, even though it has no effect; i.e. the treatment effect of acupuncture will be over-estimated.

In both of the preceding scenarios, the appropriate statistical analysis is ANCOVA adjusting for baseline scores. The first scenario corresponds to Eq. 1, with its left-hand side showing the post score as the outcome measure. The second scenario corresponds to Eq. 2, with its left-hand side showing the change score as the outcome measure.

Using change score as the outcome measure does not address the problem of RTM, nor does it take account of the baseline imbalance. Even if change score is deemed to be the appropriate outcome measure after careful consideration, ANCOVA should still be used to adjust for baseline scores, as shown by Eq. 2.

Using change score as outcome does not adjust for baseline imbalance; instead, any imbalance will be reversed due to RTM [4]. Equation 2 shows that when change score is the chosen outcome, one should still adjust for baseline using ANCOVA. In such a case, ANCOVA is the valid statistical analysis.

ANCOVA has the advantages of being unaffected by baseline imbalance [17], and it has greater statistical power than other methods [16]. An RCT reduces RTM at the design stage, but one should still use ANCOVA to adjust for baseline in the analysis stage [3].

### The validity of using change score as outcome measure

Let *r* denote the correlation between post score *Y*_1_ and baseline score *Y*_0_. Let ${s}_{Y_0}^2$ denote the sample variance of baseline score *Y*_0_, ${s}_{Y_1}^2$ denote the sample variance of post score *Y*_1_, and ${s}_{\left({Y}_1-{Y}_0\right)}^2$ denote the sample variance of the change score (*Y*_1_ − *Y*_0_). Let ${s}_{Y_0}$, ${s}_{Y_1}$, and ${s}_{\left({Y}_1-{Y}_0\right)}$ denote their corresponding standard deviations (SD).

The Appendix shows that the correlation between change score (*Y*_1_ − *Y*_0_) and baseline score *Y*_0_ is

4 $$ corr\left({Y}_1-{Y}_0,{Y}_0\right)=\frac{r\ {s}_{Y_1}-{s}_{Y_0}}{\sqrt{s_{Y_0}^2+{s}_{Y_1}^2-2\ r\ {s}_{Y_0}{s}_{Y_1}}} $$

Equation 4 shows that *orr*(*Y*_1_ − *Y*_0_, *Y*_0_) will be positive if $r>{s}_{Y_1}/{s}_{Y_0}$, and vice versa. In the special case of *r* = 0 (i.e. *Y*_1_ and *Y*_0_ are not correlated), there still will be negative correlation between change score (*Y*_1_ − *Y*_0_) and baseline score *Y*_0_. If the post score and baseline have similar variance,then *corr*(*Y*_1_ − *Y*_0_, *Y*_0_) will usually be negative because *r* ≤ 1. Most importantly, Eq. 4 shows that there is always a correlation between the change score and the baseline score; therefore, one should use ANCOVA to adjust for the baseline score.

Equation 4 was also applied when comparing methods of measurement [8], where *Y*_1_ and *Y*_0_ were replaced by test measure and standard measure, respectively. The authors of [8] show that plotting difference against standard method is misleading, because there will be a negative correlation even if the two methods are not correlated. The authors also conclude that plotting difference against the average is more useful in almost all medical measures.

We now consider the variance of the change score (*Y*_1_ − *Y*_0_). The variance sum law states that the variance of the change score is

5 $$ {s}_{\left({Y}_1-{Y}_0\right)}^2={s}_{Y_0}^2+{s}_{Y_1}^2-2\ r\ {s}_{Y_0}{s}_{Y_1} $$

Equation 5 shows that if *r* is small, ${s}_{\left({Y}_1-{Y}_0\right)}^2$ will be greater than ${s}_{Y_1}^2$; i.e. using change score will add variance compared with using post score as the outcome measure, and therefore will be less likely to show a significant result. Conversely, the post score will be more likely to show a significant result if *r* is high. However, the choice of the outcome measure should not be driven by the likelihood of a significant result; instead, it should be pre-specified in the trial protocol [17].

Using change score as outcome has undesirable implications. For example, if there is a hard lower or upper limit on the score, it may lead to “floor” or “ceiling” effects in change score. If transformation of the original scores is used during data analysis, it is not guaranteed that the transformation applies to the change score. Different transformations can reorder change scores across patients. By contrast, using post scores is always valid and never misleading [12].

The change score can be a reasonable outcome when the correlation between baseline and post scores is high (e.g. *r* > 0.8) in stable chronic conditions such as obesity [17]. In this instance, ANCOVA is still the preferred general approach.

In the Appendix, we show that the change score is always correlated with the baseline score (Eq. 9) and with the post score (Eq. 10). This is purely a statistical artefact, and it exists regardless of whether the treatment is effective or not. The method of using change score as outcome measure is prone to incorrect interpretations of such correlations.

In summary, one should be cautious about using change score as the outcome measure. If justification exists for using change score as the outcome measure, one should still adjust for baseline using ANCOVA. This will increase statistical power and avoid the pitfall of RTM.

## Discussion

### Potential imbalance of baseline in RCTs

In practice, given the finite sample size and random nature of RCTs, any important prognostic factors and baseline score may not be balanced between arms. RCTs of small or moderate sample sizes are particularly prone to such imbalances.

The balance of specific prognostic factors can be achieved during randomisation. The most commonly used randomisation methods are stratified permuted blocks [1] and minimisation [2]. Both methods allow randomisation to be stratified according to prognostic factors, such as gender, disease severity, age group, etc., which ensures that these characteristics are balanced between the treatment and control arms. One should adjust for stratification or minimisation factors during the data analysis stage [13].

However, the randomisation methods outlined above do not deal with the potential imbalance in baseline scores between arms. It is therefore of particular importance to measure the baseline scores *before* randomisation and then use ANCOVA to adjust for baseline in the data analysis stage, as shown in this paper.

### The correlation between change score and baseline score

The correlation between change score (*Y*_1_ − *Y*_0_) and baseline score *Y*_0_ has previously been observed in the context of initial blood pressure and its fall with treatment [11]. We provide a detailed mathematical derivation in the Appendix. Equation 9 shows that there is always a correlation between the change score and the baseline, regardless of any treatment effects. This correlation will be negative if *r* is small; that is, if the change score is the chosen outcome measure (for instance, of blood pressure), we will observe a fall in the blood pressure against the baseline blood pressure. An incorrect interpretation of such an observed decrease in the change score would be to conclude that the treatment is more effective for patients whose initial blood pressure is high.

### Deriving correlation between baseline score *Y*_0_ and post score *Y*_1_

In this paper, we have assumed that the value of *r*, the correlation between baseline score *Y*_0_ and post score *Y*_1_ ,is known. However, the value of *r* is usually not readily available in the design stage of an RCT.In a previous paper [9], we have shown how to derive *r* using the variance sum law based on a published paper,and then use the derived value of *r* to calculate sample size using different methods.

Once we have derived *r*, we can derive correlations between different variables using equations derived in the Appendix. In the ideal situation when the raw data are available, one can fully investigate correlations between different variables using the equations provided in this paper.

### Bland-Altman plots

The same mathematical principles derived in the Appendix can be applied to both choosing outcome measures in an RCT and assessing agreement between two measurement methods [5, 8]. A Bland-Altman plot shows the difference of the two measures on the *y*-axis and their average on the *x*-axis.

When assessing the agreement between two measurement methods, one should use a Bland-Altman plot showing the difference of the two measures against their average. The correlation *r* between the two measures does not assess their agreement [5]. We note that if the ranges of the two measures are different, their variances will be different; therefore, there will be a trend on the Bland-Altman plot, caused by the correlation shown in Eq. 13. Therefore, one should examine the variances of the two measures before using Bland-Altman plots.

Similarly, in the context of outcome measures in an RCT, one can plot the change score against the average of the baseline and post scores, as in a Bland-Altman plot. Equation 13 shows that the correlation between the change score and their average will be zero if the baseline score and post score have equal variance.

### Limitations

The methods described in this paper only consider continuous variables or outcome measures. They are not applicable to binary variables.

## Appendix

We denote the variance of variables *X* and *Y* by ${\sigma}_X^2\ \mathrm{and}\ {\sigma}_Y^2$, respectively, and their correlation *ρ*. We use *corr*(), *cov*(), *var* () as the generic notation for correlation, covariance, and variance, respectively.

### Correlation between (*Y* − *X*) and *X*

In this section, we derive *corr*(*Y* − *X*, *X*), the correlation between variables (*Y* − *X*) and *X*.

By definition, we have

6 $$ corr\left(Y-X,X\right)=\frac{\mathit{\operatorname{cov}}\left(Y-X,X\right)}{\sqrt{\mathit{\operatorname{var}}\left(Y-X\right)\cdot \mathit{\operatorname{var}}(X)}}=\frac{\mathit{\operatorname{cov}}\left(Y,X\right)-{\sigma}_X^2}{\sqrt{\mathit{\operatorname{var}}\left(Y-X\right)\cdot {\sigma}_X^2}} $$

By the variance sum law, we have

7 $$ \mathit{\operatorname{var}}\left(Y-X\right)={\sigma}_Y^2-2\rho {\sigma}_X{\sigma}_Y+{\sigma}_X^2 $$

By definition, we have

8 $$ \mathit{\operatorname{cov}}\left(Y,X\right)= corr\left(Y,X\right){\sigma}_X{\sigma}_Y=\rho {\sigma}_X{\sigma}_Y $$

Substituting Eqs. 8 and 7 into Eq. 6, we have

9 $$ corr\left(Y-X,X\right)=\frac{\rho {\sigma}_X{\sigma}_Y-{\sigma}_X^2}{\sigma_X\sqrt{\left({\sigma}_X^2-2\rho {\sigma}_X{\sigma}_Y+{\sigma}_Y^2\right)}}=\frac{\rho {\sigma}_Y-{\sigma}_X}{\sqrt{\sigma_X^2-2\rho {\sigma}_X{\sigma}_Y+{\sigma}_Y^2}} $$

### Correlation between (*Y* − *X*) and *Y*

Following the same method for deriving *corr*(*Y* − *X*, *X*) in the previous section, it can be shown that *corr*(*Y* − *X*, *Y*), the correlation between variables (*Y* − *X*) and *Y*, is

10 $$ corr\left(Y-X,Y\right)=\frac{\sigma_Y-\rho {\sigma}_X}{\sqrt{\sigma_X^2-2\rho {\sigma}_X{\sigma}_Y+{\sigma}_Y^2}} $$

Equations 9 and 10 have been discussed in the context of comparing two measurement methods [8]. In the special case of equal variance between the two methods (i.e. *σ*_*X*_ = *σ*_*Y*_), they reduce to

$$ corr\left(Y-X,X\right)=-\sqrt{\frac{1-\rho }{2}} $$

$$ corr\left(Y-X,Y\right)=\sqrt{\frac{1-\rho }{2}} $$

### Correlation between (*X* − *Y*) and (*X* + *Y*)

In this section, we derive the correlation between variables (*X* − *Y*) and (*X* + *Y*).

By definition, we have

11 $$ {\displaystyle \begin{array}{c} corr\left(X-Y,X+Y\right)=\frac{\mathit{\operatorname{cov}}\left(X-Y,X+Y\right)}{\sqrt{\mathit{\operatorname{var}}\left(X-Y\right)\cdot \mathit{\operatorname{var}}\left(X+Y\right)}}\\ {}=\frac{\sigma_X^2+\mathit{\operatorname{cov}}\left(X,Y\left)-\mathit{\operatorname{cov}}\right(Y,X\right)-{\sigma}_Y^2}{\sqrt{\mathit{\operatorname{var}}\left(X-Y\right)\cdot \mathit{\operatorname{var}}\left(X+Y\right)}}\\ {}=\frac{\sigma_X^2-{\sigma}_Y^2}{\sqrt{\mathit{\operatorname{var}}\left(X-Y\right)\cdot \mathit{\operatorname{var}}\left(X+Y\right)}}\end{array}} $$

By the variance sum law, we have

$$ \mathit{\operatorname{var}}\left(X-Y\right)={\sigma}_X^2-2{\rho \sigma}_X{\sigma}_Y+{\sigma}_Y^2 $$

$$ \mathit{\operatorname{var}}\left(X+Y\right)={\sigma}_X^2+2{\rho \sigma}_X{\sigma}_Y+{\sigma}_Y^2 $$

Therefore,

12 $$ {\displaystyle \begin{array}{c}\mathit{\operatorname{var}}\left(X-Y\right)\cdot \mathit{\operatorname{var}}\left(X+Y\right)\kern0.5em =\kern0.5em \left({\sigma}_X^2-2{\rho \sigma}_X{\sigma}_Y+{\sigma}_Y^2\right)\left({\sigma}_X^2+2{\rho \sigma}_X{\sigma}_Y+{\sigma}_Y^2\right)\\ {}=\kern0.5em {\left({\sigma}_X^2+{\sigma}_Y^2\right)}^2-{\left(2{\rho \sigma}_X{\sigma}_Y\right)}^2\end{array}} $$

Substitute Eq. 12 into Eq. 11, we have

13 $$ corr\left(X-Y,X+Y\right)=\frac{\sigma_X^2-{\sigma}_Y^2}{\sqrt{{\left({\sigma}_X^2+{\sigma}_Y^2\right)}^2-{\left(2\rho {\sigma}_X{\sigma}_Y\right)}^2}} $$

Equation 13 shows that:

- If *X* and *Y* have equal variance $\left(\mathrm{i}.\mathrm{e}.{\sigma}_X^2={\sigma}_Y^2\right)$, the correlation between (*X* − *Y*) and (*X* + *Y*) will be zero. This is true whether or not *X* and *Y* are correlated.
- If *X* and *Y* are uncorrelated (i.e. *ρ* = 0), then the correlation between (*X* − *Y*) and (*X* + *Y*) will be
  14 $$ corr\left(X-Y,X+Y\right)=\frac{\sigma_X^2-{\sigma}_Y^2}{\sigma_X^2+{\sigma}_Y^2} $$

It can be shown that

15 $$ corr\left(X-Y,X+Y\right)= corr\left(X-Y,\frac{X+Y}{2}\right) $$

A Bland-Altman plot shows the difference (i.e. *X* − *Y*) of two measures against their average $\left(\mathrm{i}.\mathrm{e}.\frac{X+Y}{2}\right)$. The right-hand side of Eq. 15 is what a Bland-Altman plot shows; therefore, what we have derived in this section also applies to Bland-Altman plots.
